# Cognitive resilience in Alzheimer’s disease: from large-scale brain networks to synapses

**DOI:** 10.1093/braincomms/fcae050

**Published:** 2024-02-16

**Authors:** Danilo Negro, Patricio Opazo

**Affiliations:** UK Dementia Research Institute, Centre for Discovery Brain Sciences, University of Edinburgh, Edinburgh Medical School, Edinburgh EH16 4SB, UK; UK Dementia Research Institute, Centre for Discovery Brain Sciences, University of Edinburgh, Edinburgh Medical School, Edinburgh EH16 4SB, UK

## Abstract

This scientific commentary refers to ‘Alteration of functional connectivity network in population of objectively-defined subtle cognitive decline’ by Zhang *et al.* (https://doi.org/10.1093/braincomms/fcae033) and ‘Posterior cingulate cortex reveals an expression profile of resilience in cognitively intact elders’ by Kelley *et al*. (https://doi.org/10.1093/braincomms/fcac162) in *Brain Communications* and ‘Determinants of cognitive and brain resilience to tau pathology: a longitudinal analysis’ by Bocancea *et al*. (https://doi.org/10.1093/brain/awad100) in *Brain*

**This scientific commentary refers to ‘Alteration of functional connectivity network in population of objectively-defined subtle cognitive decline’ by Zhang *et al.* (https://doi.org/10.1093/braincomms/fcae033) and ‘Posterior cingulate cortex reveals an expression profile of resilience in cognitively intact elders’ by Kelley *et al*. (https://doi.org/10.1093/braincomms/fcac162) in *Brain Communications* and ‘Determinants of cognitive and brain resilience to tau pathology: a longitudinal analysis’ by Bocancea *et al*. (https://doi.org/10.1093/brain/awad100) in *Brain***

## Resilience to Alzheimer’s disease

Alzheimer’s disease, the leading cause of dementia, poses a rapidly growing socio-economic burden as the global population ages. Alzheimer’s disease is characterized by the accumulation of amyloid-β plaques and tau neurofibrillary tangles alongside synaptic and neuronal loss, ultimately culminating in brain atrophy.

The relationship between Alzheimer’s disease pathology and clinical presentation is highly heterogeneous, with nearly a third of older adults displaying Alzheimer’s disease neuropathological features without cognitive impairment. The concept of cognitive reserve or resilience (CR) was introduced to account for this population, yet its biological basis remains elusive.^1^ Adding to the complexity, a paradoxical property of CR is that a delayed onset of Alzheimer’s disease symptoms is often followed by faster cognitive decline, potentially occurring at the saturation of CR.^1^ Given the limited success of disease-modifying treatments, uncovering mechanisms of resilience to Alzheimer’s disease constitutes a key priority for future therapeutic development.

**Figure 1:**
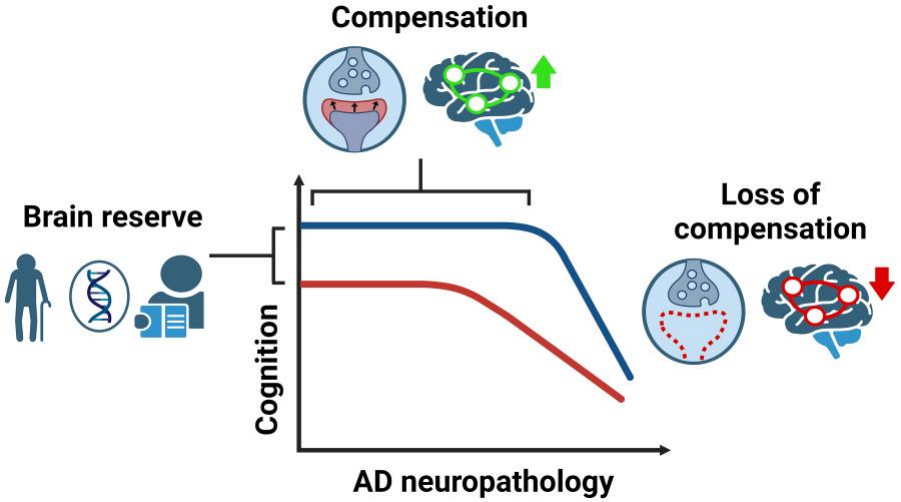
**Proposed model of CR to Alzheimer’s disease.** Brain reserve determines the baseline cognitive advantage at the onset of Alzheimer’s disease neuropathology and is influenced by factors such as age, educational attainment and genetic predisposition. Over time, compensatory mechanisms like synaptic plasticity and consequent changes in functional connectivity counteract the effect of accumulating Alzheimer’s disease neuropathology, delaying the onset of cognitive deficits. As these compensatory mechanisms are lost, cognitive decline ensues. This occurs in such a manner that the longer the delay in dementia onset due to greater compensation, the steeper the subsequent decline in cognition once these compensatory elements are lost. AD, Alzheimer’s disease. Figure was created with Biorender.com.

In this commentary, we discuss three research papers recently published in *Brain Communications* or *Brain*, investigating potential resilience mechanisms against Alzheimer’s disease across different levels, from large-scale networks to synaptic molecular processes.^2-4^ This perspective suggests that the clinical manifestation of Alzheimer’s disease may be attributed to the saturation of the brain’s compensatory capacity (Fig. 1), which novel treatments might want to enhance, possibly by targeting substrates of synaptic plasticity.

## Resilience to Alzheimer’s disease in the form of compensatory mechanisms

Epidemiological studies underscore associations between demographic/lifestyle factors, such as educational attainment, IQ and physical activity and a higher CR.^1^ These factors may confer resilience by establishing a baseline advantage in neurobiological resources (e.g. a thicker cortex and higher synaptic densities), commonly referred to as brain reserve, and/or by facilitating more dynamic compensatory processes that actively counteract the progressive loss of such resources. Longitudinal studies can distinguish whether these determinants might preserve cognition amid the accumulation of Alzheimer’s disease pathology, by providing a greater baseline brain reserve or by promoting active mechanisms of compensation over time.

Bocancea *et al.* conducted a longitudinal study in a cohort diagnosed with mild cognitive impairment or Alzheimer’s disease, investigating how demographic, biological and brain structural factors influenced the impact of accumulating tau pathology—measured with PET radiotracing—on the rate of cognitive decline and cortical thinning over time.^2^ Consistent with prior research, younger age, higher educational attainment and greater intracranial volume were associated with elevated cross-sectional cognitive and structural integrity at comparable levels of tau burden. However, while higher educational attainment and greater intracranial volume provided a baseline advantage at lower pathology levels, presumably earlier in disease progression, these factors adversely affected the trajectory of cognitive decline and cortical thinning as tau burden accumulated over time. Interestingly, younger age emerged as a mitigating factor against the impact of tau pathology on cognitive impairment and brain atrophy, both at baseline and longitudinally. This might be attributed to more preserved cellular mechanisms of compensatory plasticity in younger individuals, actively counteracting the detrimental effects of Alzheimer’s disease pathology on the rate of cognitive and structural decline.

This study reinforces the notion that compensatory mechanisms might attenuate the rate of cognitive decline in Alzheimer’s disease but paradoxically accelerate its progression once these processes are exhausted. It is worth noting that despite including cognitively impaired individuals only, by definition a population with attenuated CR, this study still indicates that protective processes might be at play at the early stages of Alzheimer’s disease. Nonetheless, the underlying biological substrate of such putative compensatory mechanisms remains elusive. A study by Zhang *et al.* suggests that resilience to Alzheimer’s disease could be implemented through compensatory changes in functional connectivity at the network level.^3^

## Compensatory changes in functional connectivity

The accumulation of Alzheimer’s disease pathology precedes the clinical onset of dementia by several decades, potentially reflecting the implementation and eventual saturation of compensatory mechanisms that contribute to CR. This temporal progression implies that individuals in a preclinical stage of Alzheimer’s disease, shortly prior to the onset of clinical dementia, may represent a population with heightened compensatory mechanisms on the verge of saturation. Subjects with objectively defined subtle cognitive decline (Obj-SCD) might correspond to such population, displaying minimal cognitive deficits that fall short of a mild cognitive impairment or dementia diagnosis and that characterize them as the earliest identifiable preclinical stage of Alzheimer’s disease.^5^ Accordingly, despite being cognitively unimpaired, Obj-SCD individuals exhibit higher rates of cognitive decline and increased Alzheimer’s disease pathology deposition compared with cognitively normal healthy agers.^3,5^

Zhang *et al.* employed resting-state functional magnetic resonance imaging to compare functional connectivity between cognitively normal, Obj-SCD and mild cognitive impairment individuals.^3^ Correlative timeseries were used to generate voxel-wise connectivity matrices, estimating brain region-specific parameters of local and global functional connectivity, degree centrality and eigenvector centrality, respectively. Obj-SCD individuals exhibited enhanced baseline local and global functional connectivity, specifically higher degree centrality in the left precuneus and eigenvector centrality in the left superior temporal gyrus, both regions involved in memory processes. Notably, these measures positively correlated with neuropsychological scores and negatively associated with blood plasma levels of the neuropathological marker neurofilament light chain across the three cohorts. This suggests that a reorganization of the connectome might constitute an early compensatory mechanism, potentially facilitating a more efficient use of remaining brain resources and/or the additional recruitment of new brain networks to maintain cognition relatively intact despite ongoing mild disease progression.

As expected from a biological mechanism potentially contributing to CR, Obj-SCD individuals paradoxically underwent a faster decline in the same functional connectivity measures over the follow-up period compared with both cognitively normal and mild cognitive impairment individuals. This reinforces the notion that this compensatory capacity can become saturated and progressively lost over time, contributing to the clinical manifestation of cognitive decline in later stages of the disease. Although changes in resting-state functional connectivity may result from various factors, a study by Kelley *et al.* suggests that processes of resilience against Alzheimer’s disease might be implemented at the synaptic level.^4^

## A synaptic perspective on resilience to Alzheimer’s disease

Identifying genes differentially expressed in the brain of cognitively unimpaired individuals with high levels of Alzheimer’s disease pathology can offer insights into the molecular and cellular basis of potential resilience mechanisms. With this aim, Kelley *et al.* conducted RNA sequencing on postmortem brain samples from cognitively resilient individuals exhibiting variable severity of tau pathology.^4^ Specifically, the analysis focused on the posterior cingulate cortex, a brain region involved in memory processes and displaying hypometabolism in Alzheimer’s disease. Excitatory pre- and post-synaptic genes were upregulated as tau pathology progressed from early (Braak I/II) to late stages (Braak III/IV) within the posterior cingulate cortex of cognitively unimpaired individuals. Notably, these upregulated synaptic genes positively correlated with better premortem cognitive performance.

Although this study lacks a direct comparison between cognitively resilient individuals and Alzheimer’s disease cases displaying similar pathology levels, a prior RNA sequencing analysis on the prefrontal cortex of subjects with Alzheimer’s disease has shown a comparable upregulation of synaptic genes in early Braak stages, which however was not maintained at later stages of tau accumulation.^6^ Altogether, these findings suggest that resilience to Alzheimer’s disease may be provided by compensatory mechanisms of plasticity at the synaptic level counteracting accumulating pathology, while the clinical manifestation of the disease may coincide with a failure in maintaining such mechanisms over time.

In summary, these studies collectively propose that longitudinal CR against accumulating Alzheimer’s disease pathology might manifest through enhanced functional connectivity in cognitively relevant brain regions, potentially mediated by a facilitation in excitatory synaptic transmission.

## Synaptic compensation in Alzheimer’s disease

Synaptic loss constitutes the best individual predictor of cognitive decline in Alzheimer’s disease,^7^ and synaptic integrity is emerging as a crucial determinant of CR against Alzheimer’s disease.^8^ In this light, potential mechanisms of compensation conferring resilience to Alzheimer’s disease are likely to involve some forms of synaptic compensatory plasticity.^9^

A potential synaptic compensatory mechanism involves the enlargement of remaining synapses, as indicated by electron microscopy studies on postmortem Alzheimer’s disease samples.^7^ Notably, the increase in average synaptic size shows a tight correlation with the extent of synaptic loss in several brain regions, such that the total synaptic area measured in these Alzheimer’s disease samples appears to be relatively similar to that of controls.^7^ An intriguing hypothesis is that the eventual loss of these enlarged compensating synapses might underlie the accelerated decline in cognitive function experienced following the onset of clinical dementia. Another emerging resilience mechanism in the context of Alzheimer’s disease is the de novo generation of new synapses. This is corroborated by longitudinal live imaging studies, providing evidence of an increase in the formation of dendritic spines in animal models of Alzheimer’s disease.^9,10^ Overall, these findings support synaptic remodelling as a resilience mechanism against Alzheimer’s disease, potentially optimizing the use of remaining brain resources in the face of accumulating pathology.

In conclusion, a comprehensive biological model for CR in Alzheimer’s disease should account for factors influencing both the delay in the onset of cognitive deficits (e.g. compensatory synaptic plasticity) and the subsequent accelerated rate of decline (e.g. loss of compensating synapses). Furthermore, it should encompass various levels, from large-scale brain network dynamics to mechanisms of synaptic compensation, as exemplified by the studies discussed here. A deeper understanding of the biological basis of resilience mechanisms in Alzheimer’s disease is crucial to harness their potential for future therapeutic development.
